# Inverted ILM flap, free ILM flap and conventional ILM peeling for large macular holes

**DOI:** 10.1186/s40942-018-0111-5

**Published:** 2018-02-19

**Authors:** Raul Velez-Montoya, J. Abel Ramirez-Estudillo, Carl Sjoholm-Gomez de Liano, Francisco Bejar-Cornejo, Jorge Sanchez-Ramos, Jose Luis Guerrero-Naranjo, Virgilio Morales-Canton, Sergio E. Hernandez-Da Mota

**Affiliations:** 1 Retina Department, Asociación para Evitar la Ceguera en Mexico, Hospital “Dr. Luis Sanchez Bulnes” IAP, Vicente García Torres #46, Col: San Lucas Coyoacán, 04030 Mexico City, DF Mexico; 2 Retina and Vitreous Department, Fundación Hospital Nuestra Señora de la Luz, Mexico City, Mexico; 3 Ophthalmology Unit, Clinica David, Morelia City, Mexico

**Keywords:** Large macular hole, Internal limiting membrane, Inverted-flap, Free-flap, Visual recovery, Surgery, Treatment

## Abstract

**Background:**

To assess closure rate after a single surgery of large macular holes and their visual recovery in the short term with three different surgical techniques.

**Methods:**

Prospective multicenter randomized controlled trial. We included treatment-naïve patients with diagnosis of large macular hole (minimum diameter of > 400 µm). All patients underwent a comprehensive ophthalmological examination. Before surgery, the patients were randomized into three groups: group A: conventional internal limiting membrane peeling, group B: inverted-flap technique and group C: free-flap technique. All study measurements were repeated within the period of 1 and 3 months after surgery. Continuous variables were assessed with a Kruskal–Wallis test, change in visual acuity was assessed with analysis of variance for repeated measurements with a Bonferroni correction for statistical significance.

**Results:**

Thirty-eight patients were enrolled (group A: 12, group B: 12, group C: 14). The closure rate was in group A and B: 91.6%; 95% CI 61.52–99.79%. In group C: 85.71%; 95% CI 57.19–98.22%. There were no differences in the macular hole closure rate between groups (*p* = 0.85). All groups improved ≈ 0.2 logMAR, but only group B reached statistical significance (*p* < 0.007).

**Conclusions:**

Despite all techniques displayed a trend toward visual improvement, the inverted-flap technique seems to induce a faster and more significant recovery in the short term.

## Background

The removal of the internal limiting membrane (ILM) has become an essential surgical step in most macular hole surgeries [1]. In skilled hands, pars plana vitrectomy with vital dye-assisted ILM peeling is a very safe and reliable procedure, which induces the closure of macular holes in up to 98% of cases [2–4]. This surgical maneuver is so successful that its indications have expanded to the surgical treatment of other macular diseases [1, 5]. However, in challenging cases like large macular holes (minimum diameter > 400 μm) and macular holes associated with high myopia, the surgical outcomes are usually poorer regardless of whether the ILM has been removed or not during surgery (closure rate ≈ 40%) [6, 7].

The retina surgeon general approach to such cases of “more is better”, has led to extensive areas of ILM denudation after surgery [8, 9]. The clinical implications of the excessive loss of ILM are not well understood, but there is evidence of anatomical changes during follow-up like progressive dissociation of optic nerve fiber layer, a decrease of the papillomacular distance and asymmetric displacement of the macula [5, 10–13]. Moreover, those large macular holes that have ended up achieving closure after conventional ILM peeling are more prone to display a V-shape, W-shape or a flat/open (flat macular hole with bare retinal pigment epithelium) closure type pattern. Despite being considered as favorable closure patterns, they are usually associated with persistent loss of photoreceptors layer (irregularities), retinal pigment epithelium defects, and foveal tissue loss that correlates with poorer visual recovery and frequent need of reoperations [14–17].

In order to improve the closure rate in complicated cases of macular holes while minimizing the possible anatomical consequences of an extensive ILM peeling; two research teams were established, one led by Michalewska et al. and second by Morizane et al., that introduced two novel surgical techniques based on the principle of ILM manipulation and conservation: the inverted flap technique and the free-flap technique [15, 16, 18]. Michalewska et al. proposed an approach in where the ILM is not completely removed, but a small remnant is left on the margin of the macular hole to cover it, while Morizane et al. proposed the creation of free ILM flap, starting from the outer border of a complete ILM peeling and then placed over the macular hole to cover it [15, 18, 19]. Both techniques aim to eliminate the anteroposterior and tangential traction exerted on the retinal surface by removing all cortical vitreous and surface components of the retina (epiretinal membranes, ILM) while stimulating cell proliferation and migration of glial cells into the macular hole. Therefore, they enhance the chances of closure and potentially improve the postoperative visual acuity [15, 16, 18].

Despite several reports supporting the efficacy of both techniques in achieving macular hole closure, well-designed studies have also reported a lack of visual recovery and persistent defect of the ellipsoid zone and photoreceptors outer segments regardless of the technique [6, 20, 21]. In addition, a good proportion of the evidence comes from studies lacking appropriate controls, randomization or blinding of the researchers, which limited the relevance of the information. Therefore, the aim of this study is to compare the single-surgery closure rate and visual outcomes of both surgical techniques in challenging cases of large macular holes against the closure rate and visual outcome of conventional 360°-ILM peeling that will serve as the control group.

## Methods

Randomized, three-center, controlled case series. The study was conducted in three different hospital centers: Asociacion Para Evitar la Ceguera en Mexico, Hospital Fundacion Nuestra Señora de la Luz and Clinica David. The study was approved by the Internal Review Board of each hospital by separate. Each review board is independent of each other and depends directly on the Mexico’s Ministry of Health. The study was conducted according to the tenets of the Declaration of Helsinki and Good clinical practice guidelines. All sensitive data were managed according to the Mexican Federal Law of Protection of Personal Data in Possession of Individuals (NOM-024-SSA3-2010), which is the local equivalent of the health insurance portability and accountability act (HIPAA) of 1996.

All patients were informed about the need and details of the surgical procedure, the possible occurrence of complications related to the surgical event, the main differences between each ILM peeling technique and the possible outcome of not doing surgery. All patients signed an informed consent form before enrollment into the study and before any measurement related to the study was done.

We included consecutive treatment-naïve patients (18 years or older), with clinical and tomographic diagnosis of Stage IV large macular holes (minimum diameter > 400 µm), regardless of gender, best-corrected visual acuity (BCVA) at presentation, time of evolution, and lens status from January 1st, 2016 to January 1st, 2017. We excluded patients with past medical history of amblyopia, diabetic retinopathy, panretinal photocoagulation, glaucoma, inflammatory eye diseases, high myopia (≥ − 6), or patients that were unable/refused to sign the informed consent form.

After enrollment, all patients underwent a comprehensive ophthalmological examination which included a complete medical history, the assessment of the BCVA in Snellen lines [later converted to its logarithm of the minimum angle of resolution, (logMAR) equivalent for statistical purposes], slit-lamp examination, fundus examination and optical coherence tomography [OCT (Spectralis HRA-OCT; Heidelberg Engineering, Heidelberg, Germany); or (Cirrus HD-OCT, Carl Zeiss Meditec, Dublin CA)].

OCT images acquired with the Cirrus HD-OCT were acquired with a 6 mm Enhanced HD 5 Raster Single Line protocol at a 0° angle and 0.25 mm spacing. Patients were asked to fixate the vision of the fellow eye on a red target placed in front of him in order to keep both eyes aligns. If the scan was not centered, the aiming beam was manually placed in the center of the macular hole, in order to ensure that the third of the 5 lines passed in the middle of the foveal defect. The third raster line was used for all measurement. Spectralis HRA-OCT images were acquired using a preset 7 Line Raster Scan of 30° × 0°, 25 frames OCT ART mean, and 240 µm spacing on high resolution. Patient protocol was similar to the Cirrus HD-OCT. If the scan was not centered, the aiming beam was manually placed in the center of the macular hole, in order to ensure that the forth of the 7 lines passed in the middle of the foveal defect. The forth raster line was used for all measurement. From each OCT study, we manually assessed them by using the caliper software tool, the minimum diameter (minimal extent of the hole), the base diameter (diameter at the level of the retinal pigment epithelium), height (maximal distance between the retinal pigment epithelium and the vitreoretinal interface) and calculated the macular hole index (MHI, ratio of the macular hole height to its base diameter).

All patients had standard three-port, sutureless, pars plana vitrectomy (PPV) and gas tamponade. The selection of the ILM peeling technique for each individual case was randomized with a Simple block randomization technique (3 × 3). The appointed ILM peeling technique was revealed to the surgeon moments before the surgery. The randomization resulted in three different groups: Patients in group A had conventional ILM peeling around the macular hole of 2 disk diameters in length at least. Patients in group B had the inverted flap technique, as described by Michalewska et al. [15, 19]. Patients in group C had the free-flap technique, either as described by Morizane et al. or Hernandez-da Mota and Bejar-Cornejo [18, 22]. All surgeries were performed by highly trained and experienced vitreoretinal surgeons (RVM, SHD, ARE and VMC) with more than 500 macular procedures each. All ILM peelings were assisted with 0.2 ml of brilliant blue G (BBG) 0.25 mg/ml, 0.025% (Sigma-Aldrich, St. Louis, MO) vital dye. PPV gauge selection (23 or 25) and tamponade selection [Sulfur Hexafluoride (SF_6_) or octafluoropropane (C_3_F_8_)] were done according to surgeon preferences with a non-expansile dilution (SF_6_: 18%, C_3_F_8_: 14%).

None of the surgeons were aware of the ILM peeling technique before the surgery. They only became aware of the appointed technique after randomization. Patients with cataract, significant enough to preclude the peeling of the ILM, had combined procedures of phacoemulsification and PPV. After surgery, all patients were instructed into a face-down position for a minimum of 3 days. Reoperations were done with a standard three-port, sutureless, pars plana air-fluid interchange, which included additional ILM staining with brilliant blue G (BBG) 0.25 mg/ml, 0.025%, enlargement of the ILM peeling and gas tamponade (18% SF_6_) and face-down position for a minimum of 3 days.

Follow-up visits were done the day after, within the period of 1 week, 1 and 3 months after surgery. During the follow-up visits, slit-lamp examination, IOP measurement and fundus examination were done by the patient’s surgeon, which was aware of the ILM peeling technique. All BCVA assessments and OCT tests during the follow-up visits were done by a different physician or technician, who was blinded to the ILM peeling technique and randomization of each patient. For analysis purpose, we only considered the baseline measurements and the 1 and 3 months follow-up visit.

Statistical analysis was done using an excel spreadsheet (Excel 2010; Microsoft Corp., Redmond, WA) and with XLSTAT application v18.06 (Addinsoft, New York, NY). General demographic data were expressed in terms of means ± standard deviations. Closure rate was reported in percentage with 95% confidence intervals for binominal distribution. The difference in the closure rate between groups was assessed in a 3 × 3 contingency table and *X*^2^ statistics. Comparison between the means of continuous independent variables among the three groups at baseline was done with a Kruskal–Wallis test; with an alpha value of 0.05 for statistical significance. Change in BCVA through time was assessed with an analysis of variance for repeated measurements, with a Bonferroni correction for the adjustment of alpha value significance. OCT parameters at baseline between groups were compared with ANOVA and Bonferroni correction, using an alpha value of 0.05 for statistical significance.

## Results

A total of 38 patients (12 men, 26 women; 17 right eyes, 21 left eyes) were enrolled into the study. At the end of the recruitment phase, 12 patients were allocated to group A; 12 into group B and 14 into group C. All patients had diagnosis of large idiopathic macular holes (minimum diameter > 400 µm). All patients enrolled into group A and B were phakic at baseline, while patients in group C, 10 were phakic and 4 were pseudophakic at baseline. The mean age of patients in group A was 61.8 ± 9.6 years, while group B was 64.2 ± 6.7 years and group C was 58.5 ± 13.2 years of age. The mean time of evolution of the macular hole at baseline for patients in group A was 3.85 ± 1.8 months, while group B was 3.06 ± 1.8 months and group C was 3.37 ± 1.3 months. There was no difference in age and time of evolution among groups (*p* = 0.58). Only one patient in group A and 1 patient in group B had an open macular hole at the end of the follow-up (closure rate of 91.67%; 95% CI 61.52–99.79%). In group C, two out of 14 patients had an open macular hole at the end of the follow-up (closure rate of 85.71%; 95% CI 57.19–98.22%). There were no differences in the macular hole closure rate between groups (*p* = 0.85). All patients were pseudophakic at the end of the surgery. The most commonly used tamponade agent was sulfur hexafluoride in 27 cases (group A: 7 cases, group B: 9 cases, group C: 11 cases). The remaining cases the surgeons used octafluoropropane as tamponade.

**Fig. 1:**
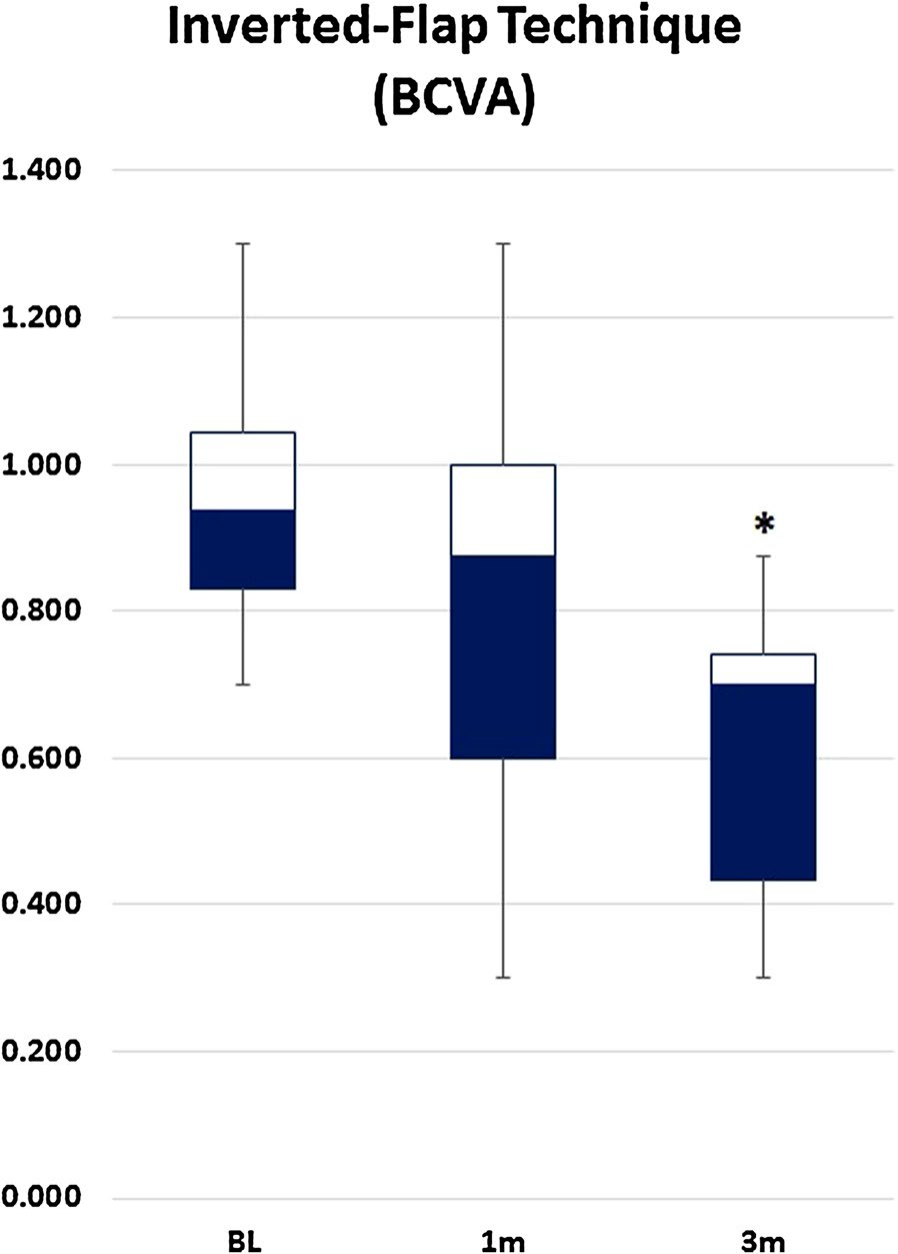
Group B change in visual acuity through time. The change reached a statistical significance at 3 months of follow-up. *BCVA* best corrected visual acuity, *BL* baseline, *m* month

The mean BCVA at baseline, 1 and 3 months of follow-up from all three groups, are summarized in Table 1. All three groups displayed a trend towards BCVA improvement (≈ 1.5 Snellen lines improvement). However, only patients in group B (inverted flap technique) had a statistically significant improvement of the BCVA at the end of the follow-up (Fig. 1). There were no differences among groups at baseline (*p* = 0.4).

**Table 1:** Change in BCVA through the study

Surgical technique	Baseline (logMAR)	1 month (logMAR)	3 months (logMAR)	Alpha (3 months)
Group A				
(Conventional 360° ILM peeling)	0.925 ± 0.5	0.656 ± 0.06	0.707 ± 0.3	0.3
Group B				
(Inverted-flap technique)	0.953 ± 0.2	0.794 ± 0.3	0.616 ± 0.2	0.007*
Group C				
(Free-flap technique)	1.097 ± 0.4	0.951 ± 0.4	0.704 ± 0.3	0.06
Alpha among groups	0.6	0.6	0.1	

All three groups displayed a trend toward visual improvement, but only patients in group B reached statistical significance at the 3 months of follow-up

*BCVA* best corrected visual acuity, *ILM* internal limiting membrane

*Statistically significant

OCT parameters at baseline are summarized in Table 2. In general, all patients displayed similar characteristics by OCT, with no statistical significant difference at baseline measurements. The MHI (a predictor factor for macular hole closure), did not show a significant difference among the groups (group A: 0.42, group B: 0.47, group C: 0.47, *p* = 0.9).

**Table 2:** Macular hole measurements at baseline

Surgical technique	Minimum	Base (µm)	Height (µm)	MHI (µm)
Group A				
(Conventional 360° ILM peeling)	522.22 ± 82.73	952.5 ± 84.9	396.75 ± 50.52	0.42 ± 0.06
Group B				
(Inverted-flap technique)	608.89 ± 213	993.67 ± 484.21	463.44 ± 121.82	0.47 ± 1.01
Group C				
(Free-flap technique)	643.83 ± 162.74	1020.67 ± 212.46	463.17 ± 94.78	0.47 ± 0.12
Alpha	0.3	0.9	0.4	0.9

*Minimum* minimum diameter, *Base* base diameter, *Height* macular hole height, *MHI* macular hole index, *ILM* internal limiting membrane

**Fig. 2:**
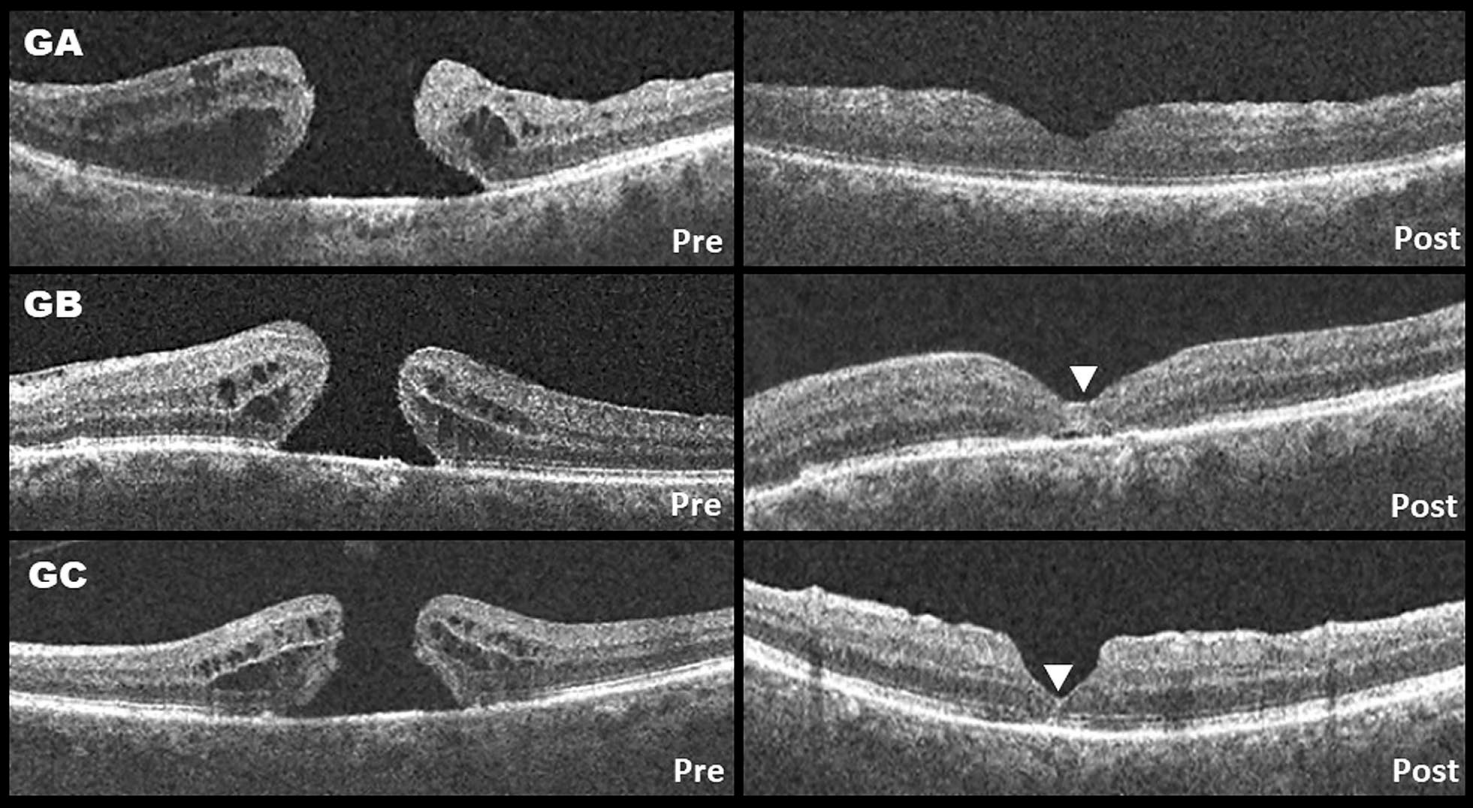
Representative cases from each group. The “pre” image is at baseline, the “post” image is at the 3 months follow-up. GA: Conventional 360 ILM peeling, displaying an asymmetrical U-shape closure configuration. In GB and GC (group B and C) the white arrowhead points to a hyperreflective area within the macular hole that may suggest retinal gliosis. The area is wider in the inverted-flap technique group. GB displays a symmetrical U-shape closure configuration. GC displays a symmetrical V-shape closure configuration

Regarding the closure patterns: six patients in group A displayed a U-shape and 5 displayed a V-shape closure type. In group B, 7 patients displayed a U-shape closure and 4 displayed a V-shape closure type. There were no W-shape closures or flat-open macular holes in group A and B (Fig. 2). Two patients (one in each group) had an open macular hole at the end of the 3 months follow-up. Both patients underwent a second procedure which included additional ILM peeling and gas tamponade. Both macular holes were closed after the second procedure. In group C, 7 patients displayed a U-shape, 5 displayed a V-shape closure type and 2 remained open after 3 months follow-up. The two patients in group C with an open macular hole underwent a second procedure, similar to patients in group A and B with open macular holes. One patient displayed a flat-open configuration after the second surgery and the other remained open despite reoperation.

## Discussion

The staining and removal of the ILM in cases of macular hole have been widely adopted and evolved since its original description in 1991 and it is considered by the majority of retinal specialist as the standard of care [23–25]. Despite no definitive evidence supporting a significant visual gain due to the sole action of removing the ILM; the evidence does suggest an increased rate of macular hole closure, decreased rate of reopening and decreased rate of reoperations [26]. However, the surgical management of large macular holes have proven to be challenging even with ILM peeling. The surgery of such cases may end in large areas of ILM-denudated retina (sometimes the entire macula); as an extreme effort to increase tissue flexibility while increasing the chance of a primary closure (“more is better”) [6, 8, 9, 27]. The physiological repercussions of such approach have not been fully studied. But there are evidence of anatomical consequences like Müller cell damage and shrinkage, asymmetrical displacement of the macula (inferior and nasal), thinning of the temporal retina, decrease of the distance between the fovea and the optic nerve, dissociation of the optic nerve fiber layer and micro scotomas [5].

The approach proposed by Michalewska et al. and Morizane et al. incorporates the novel concept of inducing retinal gliosis from within the macular hole by using the remnants of the ILM (serves as a scaffold), as a means of enhancing closure. In addition to eliminating all tractional force from the surface of the retina, this approach allows more ILM conservation and potentially decreasing some of the physiological consequences of extensive ILM-removal [15, 18, 28].

On her original description of the inverted-flap technique, Michalewska et al. reported a 98% closure rate of large macular holes (> 400 µm) and only 2% of the closure patterns were flat-open configurations [15, 16]. After 12 months of follow-up, the vision was significantly better in eyes without the inverted flap technique (0.7 vs 0.28 logMAR, *p* < 0.01) [15, 16]. It is important to note that Michalewska originally described the technique using air instead of gas, used trypan blue for ILM staining and defined anatomical success as the disappearance of the macular hole or foveal defect of neurosensory retina with flattened cuff of retina edema around the hole [15]. Other authors like Kuriyama et al., Chen et al. and Kase et al. also reported high success rate with this technique with follow-ups ranging from 1 month to 1 year [6, 29]. Although all authors agreed that there is a trend towards visual improvement with this technique, most of the case series failed to demonstrate a statistically significant visual improvement at the end of the follow-up. The reason for this observation is not well understood. However, authors like Baba et al. and Kase et al., have pointed to a possible toxicity effect due to the long lasting contact of the ILM (stained with trypan blue or indocyanine green) with the retina and retinal pigment epithelium [6, 20, 30]. Small series have suggested that the use of Brilliant blue G as the staining agent may lead to better visual outcomes [16, 20, 30]. In a retrospective analysis of her own cases, Michalewska et al. recognized that the development of a U-shape closure type was the most prevalent after the inverted-flap technique and had better functional prognosis (2 lines improvement) than other types of closure [17]. The postoperative structural analysis of the fovea demonstrated that patients with U-shape closure had smaller photoreceptor layer defects (linear defect, volumetric defect, inner segment/outer segment junction abnormalities) and normal retinal thickness at the end of the follow-up [11, 17, 31]. In cases in were only a thin ILM-flap (flap-closure) was noted over the macular hole after the surgery, regeneration of retinal tissue starting from the external limiting membrane, followed by restoration of the ellipsoids zone layer was observed during the following months [17, 31].

Regarding the free flap technique, Morizane et al. reported a closure rate of large macular holes of 90% on a small case series of ten patients [18]. They did observe a significant improvement in visual acuity of 0.2 logMAR in at least 8 patients (*p* < 0.007) after a follow-up of 12 months [18]. The proper placement and frequent dislodgement of the free flap during the air-fluid exchange or during the postoperative days have been a recurrent issue with this technique. Therefore minor modifications to the original technique using different agents like perfluorocarbon liquids for free flap placement, viscoelastic plugs or autologous serum as tissue adhesives have been proposed basically with the same level of anatomical success [32–34]. In addition to retinal gliosis, Morizane et al. discussed the potential role of ILM peeling with the physical placement of a free-flap inside the macular hole. The benefits could include; dedifferentiation of cells and photoreceptor reposition, photoreceptor migration into the retina defect and proliferation of Müller cells [18].

However, it is possible that the functional outcome after a macular hole surgery is more related to the preoperative foveal tissue damage and the morphological variations of and individual’s fovea than to the surgical technique [35]. In a recent review, Chung and Byeon described two “critical events” during the formation and progression of a macular holes: anteroposterior vitreous traction causing breaks in both the ILM and external limiting membrane and edema of the macular hole border due to hydration of the retina, which develops after the outer retina is directly exposed to vitreous fluid [35]. The structural analysis of the foveal damage by OCT after the anteroposterior vitreous traction can be divided into two morphological different types: type A or dehiscent type and type B or tearing type. Each of them can be paired with a specific macular hole stage according to Gass classification. In type A, fewer outer foveal tissue is loss during the anteroposterior traction. Therefore, more Müller cells are preserved [35]. In large and chronic macular holes, the centripetal movement of the retina induced by the gas bubble may not be sufficient to promote closure. Therefore, the placement of an inverted-flap or a free flap over the macular defect will reduce further hydration and eversion of the retina tissue, promoting glial proliferation [31, 35]. In addition, in type A macular holes, the increase elasticity of the ganglion cell layer and inner nuclear layer due to the unfolding of the preserved foveal Müller cells (Z-shaped, “atypical” Müller cells) is responsible for the centripetal movement of the retina toward the fovea rather than glial proliferation. This remodeling of the foveal tissue promotes the immediate apposition of the external limiting membrane after surgery, which results in a better foveal contour and higher postoperative visual acuities. In a type B macular hole, more Müller cells are loss during the anteroposterior vitreous traction. Therefore, the regeneration of the foveal tissue is more difficult, resulting in persistent thick foveola and small foveal avascular zone [35].

In our study, we conducted a prospective analysis comparing the inverted flap-technique, the free-flap technique, and the conventional 360° ILM peeling. We assessed the anatomical success in terms of closure rate after a single surgery and functional success in terms of change in visual acuity. Our results showed that the inverted-flap technique and the traditional 360° ILM peeling had a slightly higher closure rate than the free-flap technique (91 vs 85%; *p* = 0.85). However, it is important to point out that due to the small number of participants in all three groups, the 95% confidence intervals are wide and can easily overlap with each other. Regarding the closure rate after a single surgery of the inverted flap technique and the free-flap technique, our results are similar to those published so far, with high closure rate (above 80%). Nevertheless, conversely to the work by Michalewska et al. and Morizane et al., our prospective study did not find any difference with the conventional 360°-ILM peeling, which also displayed a high closure rate.

Regarding BCVA, all three techniques displayed a trend toward the improvement of at least 0.1–0.4 logMAR (as described previously in other studies). The small sample and wide variability could have been prevented to reach statistical significance. However, even with these limitations and in contrast with previous reports, patients on the inverted-flap technique had a significant visual gain at 3 months follow-up against the baseline value, with a highly significant *p* value (0.007) and adjusted with the Bonferroni correction. It is important to note that all three groups had basically the same amount of favorable macular hole closure patterns (U-shape) per group and all ILM staining were done with Brilliant blue G (less risk for toxicity). Unfortunately, a finer structural analysis of the OCT during the follow-up is lacking and we couldn’t assess ellipsoid zone or photoreceptor recovery. While it is impossible to predict if the other two groups will eventually gain statistical significance after a longer follow-up time with the current study design; it is possible that the BCVA in all groups keep showing signs of improvement up to 6 months after surgery.

In addition to the limitation of a small sample size, lack of sample calculation and short follow-up, the study has other limitations that we will like to address. Although we did considerable efforts to standardize the surgical procedure, the ultimate decision regarding the type of gas tamponade was made according to the surgeon’s preference and not by strict surgical protocol. The immediate consequence is that our sample can be divided further into two separate groups: those with short acting tamponades (sulfur hexafluoride), which made the majority of cases (71%) and those with longer acting tamponades (octafluoropropane). There is no conclusive evidence that favors the use of a longer acting gas over a short acting gas in cases of macular holes [36, 37]. In a study by Modi et al., the author found no significant difference in terms of anatomical closure rate, final visual acuity improvement and postoperative complications (rise of intraocular pressure, incidence of glaucoma and visually significant cataract formation) between sulfur hexafluoride and octafluoropropane, regardless of stage, size and duration of the hole [38]. Similar observations regarding the lack of difference in terms of closure rate has been reported by Kim et al., Jackson et al. and Briand et al. on their respective series [39–41]. Nevertheless, the selection of a longer acting gas tamponade can delay visual recovery for 4–6 weeks after the surgery. Therefore, due to the short follow-up in our series, the assessment of visual recovery should be taken with caution, since it may have been skewed by the slow gas absorption in up to 29% of the cases.

Another possible limitation is the use of two different OCT platforms in our study. The Cirrus HD-OCT and the Spectralis HRA-OCT have each a different proprietary internal software algorithms for tissue segmentation and measurement [42]. Wolf-Schnurrbusch et al. and Mylonas et al. compared the measurement of central retinal thickness among several different OCT devices [43, 44]. Although they found good correlation between all devices, results from Cirrus HD-OCT and Spectralis HRA-OCT tended to be higher than the rest, being the measurements from Spectralis HRA.OCT higher than the Cirrus HD-OCT [43, 44]. This differences have been found to be significant by Smretschnig et al., especially in the presence of macular pathology [42]. However, this source of bias is significant only when using and comparing the automatic software segmentation from this two different devices. In our study we did not use this feature. We relied on manual measurements by expert operators Instead, and acquired our measurements by using the caliper software tool available each OCT machine. This introduces a different type of bias, since the results are highly dependent on operator expertise and tissue appreciation. Since we did not have a central reading center or assessed the interobserver variability among our centers, our results are subject to human error and must be taken with caution.

## Conclusions

In summary, large macular holes (minimum diameter > 400 µm) are surgical challenges with poorer than usual anatomical prognosis. The inverted-flap and free-flap technique are surgical alternatives that may improve the chances of a better anatomical outcome. Our results showed no difference between this techniques and conventional (360°) ILM peeling after a single surgery. However, patients allocated into the inverted-flap group showed a trend to have faster visual recovery that might be significant in the short term (3 months of follow-up). Nevertheless, the study has considerable bias due to short follow-up, small sample size and the possibility that a slow-acting gas could have interfere with the 1-month BCVA assessment. A study with a larger sample and longer follow-up is needed in order to corroborate this observation and to assess if the visual changes are sustained over time.

## Abbreviations

ILM: Internal limiting membrane
HIPAA: Health insurance portability and accountability act
BCVA: Best-corrected visual acuity
logMAR: Logarithm of the minimum angle of resolution
OCT: Optical coherence tomography
MHI: Macular hole index
PPV: Pars plana vitrectomy
BBG: Brilliant blue G

## References

[1] Almony A, Nudleman E, Shah GK, Blinder KJ, Eliott DB, Mittra RA, Tewari A (2012). Techniques, rationale, and outcomes of internal limiting membrane peeling. Retina.

[2] Beutel J, Dahmen G, Ziegler A, Hoerauf H (2007). Internal limiting membrane peeling with indocyanine green or trypan blue in macular hole surgery: a randomized trial. Arch Ophthalmol.

[3] Chung CY, Wong DS, Li KK (2015). Is it necessary to cover the macular hole with the inverted internal limiting membrane flap in macular hole surgery? A case report. BMC Ophthalmol.

[4] Andrew N, Chan WO, Tan M, Ebneter A, Gilhotra JS (2016). Modification of the inverted internal limiting membrane flap technique for the treatment of chronic and large macular holes. Retina.

[5] Morescalchi F, Costagliola C, Gambicorti E, Duse S, Romano MR, Semeraro F (2017). Controversies over the role of internal limiting membrane peeling during vitrectomy in macular hole surgery. Surv Ophthalmol.

[6] Kase S, Saito W, Mori S, Saito M, Ando R, Dong Z, Suzuki T, Noda K, Ishida S (2017). Clinical and histological evaluation of large macular hole surgery using the inverted internal limiting membrane flap technique. Clin Ophthalmol.

[7] Hussain N, Hussain A (2016). Successful closure of treatment-naive, flat edge (Type II), full-thickness macular hole using inverted internal limiting membrane flap technique. Int Med Case Rep J.

[8] Wong D, Steel DH (2016). Free ILM patch transplantation for recalcitrant macular holes; should we save some internal limiting membrane for later?. Graefes Arch Clin Exp Ophthalmol.

[9] Goker YS, Koc M, Yuksel K, Yazici AT, Demir A, Gunes H, Ozpinar Y (2016). Relationship between peeled internal limiting membrane area and anatomic outcomes following macular hole surgery: a quantitative analysis. J Ophthalmol.

[10] Ito Y, Terasaki H, Takahashi A, Yamakoshi T, Kondo M, Nakamura M (2005). Dissociated optic nerve fiber layer appearance after internal limiting membrane peeling for idiopathic macular holes. Ophthalmology.

[11] Michalewska Z, Michalewski J, Nawrocki J (2010). Continuous changes in macular morphology after macular hole closure visualized with spectral optical coherence tomography. Graefes Arch Clin Exp Ophthalmol.

[12] Pak KY, Park KH, Kim KH, Park SW, Byon IS, Kim HW, Chung IY, Lee JE, Lee SJ, Lee JE (2017). Topographic changes of the macula after closure of idiopathic macular hole. Retina.

[13] Ishida M, Ichikawa Y, Higashida R, Tsutsumi Y, Ishikawa A, Imamura Y (2014). Retinal displacement toward optic disc after internal limiting membrane peeling for idiopathic macular hole. Am J Ophthalmol.

[14] Kang SW, Ahn K, Ham DI (2003). Types of macular hole closure and their clinical implications. Br J Ophthalmol.

[15] Michalewska Z, Michalewski J, Adelman RA, Nawrocki J (2010). Inverted internal limiting membrane flap technique for large macular holes. Ophthalmology.

[16] Michalewska Z, Michalewski J, Dulczewska-Cichecka K, Adelman RA, Nawrocki J (2015). Temporal inverted internal limiting membrane flap technique versus classic inverted internal limiting membrane flap technique: a comparative study. Retina.

[17] Michalewska Z, Michalewski J, Cisiecki S, Adelman R, Nawrocki J (2008). Correlation between foveal structure and visual outcome following macular hole surgery: a spectral optical coherence tomography study. Graefe’s archive for clinical and experimental ophthalmology = Albrecht von Graefes Archiv fur klinische und experimentelle Ophthalmologie.

[18] Morizane Y, Shiraga F, Kimura S, Hosokawa M, Shiode Y, Kawata T, Hosogi M, Shirakata Y, Okanouchi T (2014). Autologous transplantation of the internal limiting membrane for refractory macular holes. Am J Ophthalmol.

[19] Michalewska Z, Michalewski J, Dulczewska-Cichecka K, Nawrocki J (2014). Inverted internal limiting membrane flap technique for surgical repair of myopic macular holes. Retina.

[20] Sasaki H, Shiono A, Kogo J, Yomoda R, Munemasa Y, Syoda M, Otake H, Kurihara H, Kitaoka Y, Takagi H (2017). Inverted internal limiting membrane flap technique as a useful procedure for macular hole-associated retinal detachment in highly myopic eyes. Eye (Lond).

[21] Chen SN, Yang CM (2016). Inverted internal limiting membrane insertion for macular hole-associated retinal detachment in high myopia. Am J Ophthalmol.

[22] Hernandez-da Mota SE, Bejar-Cornejo F (2016). Modified technique of autologous transplantation of internal limiting membrane for macular hole. Cir Cir.

[23] Chen Z, Zhao C, Ye JJ, Wang XQ, Sui RF (2016). Inverted internal limiting membrane flap technique for repair of large macular holes: a short-term follow-up of anatomical and functional outcomes. Chin Med J (Engl).

[24] Kelly NE, Wendel RT (1991). Vitreous surgery for idiopathic macular holes. Results of a pilot study. Arch Ophthalmol.

[25] Kadonosono K, Itoh N, Uchio E, Nakamura S, Ohno S (2000). Staining of internal limiting membrane in macular hole surgery. Arch Ophthalmol.

[26] Spiteri Cornish K, Lois N, Scott N, Burr J, Cook J, Boachie C, Tadayoni R, la Cour M, Christensen U, Kwok A (2013). Vitrectomy with internal limiting membrane (ILM) peeling versus vitrectomy with no peeling for idiopathic full-thickness macular hole (FTMH). Cochrane Database Syst Rev.

[27] Matsumura T, Takamura Y, Tomomatsu T, Arimura S, Gozawa M, Kobori A, Inatani M (2016). Comparison of the inverted internal limiting membrane flap technique and the internal limiting membrane peeling for macular hole with retinal detachment. PLoS ONE.

[28] Moisseiev E, Yiu G (2016). Role of tractional forces and internal limiting membrane in macular hole formation: insights from intraoperative optical coherence tomography. Case Rep Ophthalmol.

[29] Kuriyama S, Hayashi H, Jingami Y, Kuramoto N, Akita J, Matsumoto M (2013). Efficacy of inverted internal limiting membrane flap technique for the treatment of macular hole in high myopia. Am J Ophthalmol.

[30] Baba T, Hagiwara A, Sato E, Arai M, Oshitari T, Yamamoto S (2012). Comparison of vitrectomy with brilliant blue G or indocyanine green on retinal microstructure and function of eyes with macular hole. Ophthalmology.

[31] Boninska K, Nawrocki J, Michalewska Z (2017). Mechanism of “Flap Closure” after the inverted internal limiting membrane flap technique. Retina..

[32] Hernandez-da Mota SE, Bejar-Cornejo F (2016). Modified technique of autologous transplantation of internal limiting membrane for macular hole. Cir Cir.

[33] Song Z, Li M, Liu J, Hu X, Hu Z, Chen D (2016). Viscoat assisted inverted internal limiting membrane flap technique for large macular holes associated with high myopia. J Ophthalmol.

[34] Park SW, Pak KY, Park KH, Kim KH, Byon IS, Lee JE (2015). Perfluoro-*n*-octane assisted free internal limiting membrane flap technique for recurrent macular hole. Retina.

[35] Chung H, Byeon SH (2017). New insights into the pathoanatomy of macular holes based on features of optical coherence tomography. Surv Ophthalmol.

[36] Madi HA, Masri I, Steel DH (2016). Optimal management of idiopathic macular holes. Clinical ophthalmology.

[37] Tam ALC, Yan P, Gan NY, Lam WC (2018). The current surgical management of large, recurrent, or persistent macular holes. Retina..

[38] Modi A, Giridhar A, Gopalakrishnan M (2017). Sulfurhexafluoride (Sf6) versus perfluoropropane (C3f8) gas as tamponade in macular hole surgery. Retina.

[39] Jackson TL, Donachie PH, Sparrow JM, Johnston RL (2013). United Kingdom National Ophthalmology Database study of vitreoretinal surgery: report 2, macular hole. Ophthalmology.

[40] Kim SS, Smiddy WE, Feuer WJ, Shi W (2008). Outcomes of sulfur hexafluoride (SF6) versus perfluoropropane (C3F8) gas tamponade for macular hole surgery. Retina.

[41] Briand S, Chalifoux E, Tourville E, Bourgault S, Caissie M, Tardif Y, Giasson M, Boivin J, Blanchette C, Cinq-Mars B (2015). Prospective randomized trial: outcomes of SF(6) versus C(3)F(8) in macular hole surgery. Can J Ophthalmol.

[42] Smretschnig E, Krebs I, Moussa S, Ansari-Shahrezaei S, Binder S (2010). Cirrus OCT versus spectralis OCT: differences in segmentation in fibrovascular pigment epithelial detachment. Graefe’s archive for clinical and experimental ophthalmology = Albrecht von Graefes Archiv fur klinische und experimentelle Ophthalmologie.

[43] Wolf-Schnurrbusch UE, Ceklic L, Brinkmann CK, Iliev ME, Frey M, Rothenbuehler SP, Enzmann V, Wolf S (2009). Macular thickness measurements in healthy eyes using six different optical coherence tomography instruments. Investig Ophthalmol Vis Sci.

[44] Mylonas G, Ahlers C, Malamos P, Golbaz I, Deak G, Schuetze C, Sacu S, Schmidt-Erfurth U (2009). Comparison of retinal thickness measurements and segmentation performance of four different spectral and time domain OCT devices in neovascular age-related macular degeneration. Br J Ophthalmol.

